# Adenosine receptor agonism protects against NETosis and thrombosis in antiphospholipid syndrome

**DOI:** 10.1038/s41467-019-09801-x

**Published:** 2019-04-23

**Authors:** Ramadan A. Ali, Alex A. Gandhi, He Meng, Srilakshmi Yalavarthi, Andrew P. Vreede, Shanea K. Estes, Olivia R. Palmer, Paula L. Bockenstedt, David J. Pinsky, Joan M. Greve, Jose A. Diaz, Yogendra Kanthi, Jason S. Knight

**Affiliations:** 1 Division of Rheumatology, Department of Internal Medicine, University of Michigan, Ann Arbor, MI 48109 USA; 2 Department of Biomedical Engineering, University of Michigan, Ann Arbor, MI 48109 USA; 3 Division of Hematology and Oncology, Department of Internal Medicine, University of Michigan Medical School, Ann Arbor, MI 48109 USA; 4 Department of Molecular and Integrative Physiology, University of Michigan, Ann Arbor, MI 48109 USA; 5 Division of Cardiovascular Medicine, Department of Internal Medicine, University of Michigan, Ann Arbor, MI 48109 USA; 6 Department of Vascular Surgery, University of Michigan, Ann Arbor, MI 48109 USA; 7 Division of Cardiology, Ann Arbor Veterans Administration Healthcare System, Ann Arbor, MI 48109 USA

**Keywords:** Autoimmunity, Neutrophils, Thrombosis, Antiphospholipid syndrome

## Abstract

Potentiation of neutrophil extracellular trap (NET) release is one mechanism by which antiphospholipid antibodies (aPL Abs) effect thrombotic events in patients with antiphospholipid syndrome (APS). Surface adenosine receptors trigger cyclic AMP (cAMP) formation in neutrophils, and this mechanism has been proposed to regulate NETosis in some contexts. Here we report that selective agonism of the adenosine A_2A_ receptor (CGS21680) suppresses aPL Ab-mediated NETosis in protein kinase A-dependent fashion. CGS21680 also reduces thrombosis in the inferior vena cavae of both control mice and mice administered aPL Abs. The antithrombotic medication dipyridamole is known to potentiate adenosine signaling by increasing extracellular concentrations of adenosine and interfering with the breakdown of cAMP. Like CGS21680, dipyridamole suppresses aPL Ab-mediated NETosis via the adenosine A_2A_ receptor and mitigates venous thrombosis in mice. In summary, these data suggest an anti-inflammatory therapeutic paradigm in APS, which may extend to thrombotic disease in the general population.

## Abstract (web summary)

Antiphospholipid syndrome is characterised by increased neutrophil extracellular trap formation (NETosis) and, consequently, increased thrombotic events. Here Ali et al. show that treatment with adenosine receptor agonists suppresses NETosis and venous thrombosis in mouse models of antiphospholipid syndrome.

## Introduction

Thrombotic events are among the leading causes of morbidity in patients with lupus. Antiphospholipid antibodies, present in one-third of lupus patients, are a major driver of this risk and help define a complication known as antiphospholipid syndrome (APS). Patients are classified as having APS when circulating antiphospholipid antibodies are detected in the setting of certain criteria-fulfilling events, including deep vein thrombosis, pulmonary embolism, stroke, and myocardial infarction^1^. The diagnosis of APS is not limited to patients with lupus and more than half of patients (53% in the largest study to date) are diagnosed as a standalone syndrome—primary APS^2^. Overall, APS is felt to be the leading acquired cause of thrombosis in the United States^3^. APS also places patients at increased risk for pregnancy loss, cytopenias, cardiac valve lesions, and neurologic complications^4^. Patients with APS are typically treated with anticoagulants such as warfarin, which are not always effective in preventing thrombosis, and at the same time offer no protection against the varied non-thrombotic manifestations of APS, such as cardiac valve damage, thrombocytopenia, and cognitive decline^5^. Interestingly, the best described antigen in APS is not a phospholipid, but rather a phospholipid-binding protein, beta-2 glycoprotein I (β_2_GPI). This cationic lipid-binding protein is made especially by the liver and is found in plasma at 50–200 µg ml^−1^ ^6,7^. While the pathophysiology of APS has yet to be fully elucidated, the prevailing opinion currently is that anti-β_2_GPI antibodies (and in some cases antibodies to other, similar phospholipid-binding proteins) potentiate thrombosis by engaging their antigen on cell surfaces, and thereby promoting the activation of endothelial cells, platelets, monocytes, and neutrophils^8–10^.

Despite the abundance of human neutrophils (100 billion new cells produced each day), our knowledge of their role—and malleability—in chronic disease remains quite rudimentary. Our group has recently undertaken the study of neutrophils in patients with autoimmune thrombophilia, using APS as the model disease. We have revealed that the neutrophils of APS patients have a reduced threshold for the release of neutrophils extracellular traps (NETs)—prothrombotic tangles of DNA, histones, and granule-derived proteins expelled from dying neutrophils^11^. We have also demonstrated that purified control neutrophils display β_2_GPI—a key APS autoantigen—on their surface, and can be triggered to release NETs in TLR4-dependent fashion by exposure to anti-β_2_GPI isolated from APS patients^11^. Using a human/mouse chimeric model, we have shown that NETs are required for APS-potentiated thrombosis^12^. By profiling the transcriptome of APS neutrophils, we have discovered a strikingly activated signature in APS neutrophils as compared with controls^13^, which has revealed previously unconsidered therapeutic targets in APS such as P-selectin glycoprotein ligand-1 (PSGL-1).

Recent evidence suggests that the second messenger cyclic AMP (cAMP) may suppress NETosis in some contexts^14,15^. Here, we hypothesized that surface adenosine receptors (which trigger cAMP formation in neutrophils) may serve as an endogenous counterpoint to thromboinflammatory disease, and that pharmacological agonism of adenosine receptors may therefore mitigate the thrombotic manifestations of APS. Adenosine receptors vary in both affinity for adenosine and tissue/cell distribution^16^. All four adenosine receptors have been described on neutrophils^17^. The adenosine A_1_ receptor, which has a relatively high affinity for adenosine, promotes neutrophil chemotaxis. In contrast, the other three adenosine receptors, which may only become activated when the local environment is flooded with excess adenosine, tend to silence neutrophils^16,17^. In particular, the A_2A_ and A_3_ receptors are expressed at high levels on neutrophils, where they suppress neutrophil effector functions such as reactive oxygen species (ROS) formation when activated^17–19^. A single study has demonstrated that agonism of the A_2A_ receptor is protective against nephritis in a model of lupus^20^, and work by our group has shown that deletion of the ectonucleotidase CD73 (which plays a key role in generating extracellular adenosine) exacerbates endothelial dysfunction in lupus mice^21^. However, the specific impact of adenosine signaling on lupus-relevant vascular disease and neutrophil activity, as well as the role of adenosine receptors in APS, are heretofore unexplored. We now report that a specific adenosine receptor agonist attenuates aPL Ab-mediated NETosis in vitro and venous thrombosis in mice. Furthermore, the antithrombotic medication dipyridamole phenocopies adenosine receptor agonism in terms of suppressing both NETosis and venous thrombosis.

## Results

### cAMP suppresses NETosis triggered by APS IgG

**Fig. 1:**
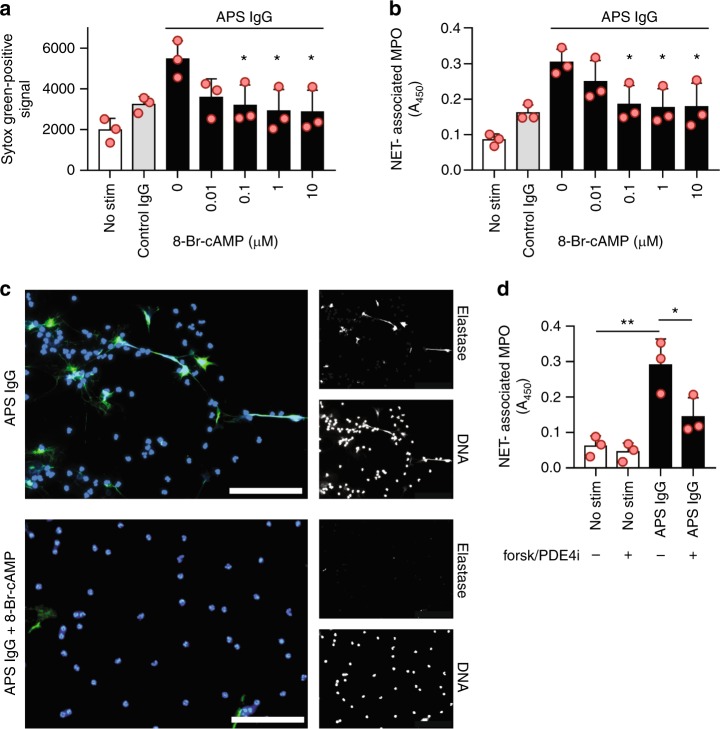
Cyclic AMP (cAMP) suppresses NETosis in response to APS IgG. Neutrophils were isolated from healthy volunteers and then treated with either control IgG or APS IgG (pooled from four patients with primary APS) for 3 h. Some samples were additionally treated with 8-Br-cAMP at various concentrations. In panel **a**, total extracellular DNA was measured as relative fluorescence units upon the addition of Sytox Green. In panel **b**, an independent set of experiments was performed, with NETosis quantified by measuring the enzymatic activity of nuclease-liberated myeloperoxidase (MPO). Mean and standard deviation are presented for *n* = 3 independent experiments; *p < 0.05 as compared with the 0 µM group by one-way ANOVA corrected with Dunnett’s test. **c** NETosis was assessed qualitatively by immunofluorescence microscopy. 8-Br-cAMP = 1 µM, blue = DNA, green = extracellular neutrophil elastase, and scale bar = 100 microns. **d** Neutrophils were treated with either control IgG or APS IgG. Forskolin and PDE4 inhibitor were additionally added to some samples. NETosis was quantified by the enzymatic activity of nuclease-liberated MPO; *p < 0.05 and **p < 0.01 by one-way ANOVA corrected with Sidak’s test

Antiphospholipid antibodies potentiate NETosis and thereby promote thrombotic events^11–13^. Recent evidence suggests that the second messenger cAMP suppresses NETosis in some contexts^14,15^. As both the formation and degradation of cAMP are amenable to pharmacologic manipulation, we reasoned that this might be an attractive pathway to target in APS neutrophils. 8-Br-cAMP is a cell-permeable, degradation-resistant analogue of cAMP. We found that treatment of neutrophils with 8-Br-cAMP mitigated the release of extracellular DNA (Sytox Green-positive signal, Fig. 1a) and NETs (Fig. 1b) by control neutrophils that were activated with IgG isolated and pooled from four primary APS patients (APS IgG). The clinical characteristics of these patients are described in Supplementary Table 1. NETosis was also assessed qualitatively by immunofluorescence microscopy with similar results (Fig. 1c). Given these data, we reasoned that similar suppression of NETosis should be achieved with activation of endogenous cAMP via the adenylyl cyclase activator forskolin^14^. Indeed, the combination of forskolin and a phosphodiesterase 4 inhibitor mitigated NETosis (Fig. 1d). These data reinforce our hypothesis that manipulation of cAMP levels may be an effective way to mitigate NETosis in APS.

### Agonism of the adenosine A_2A_ receptor suppresses NETosis

**Fig. 2:**
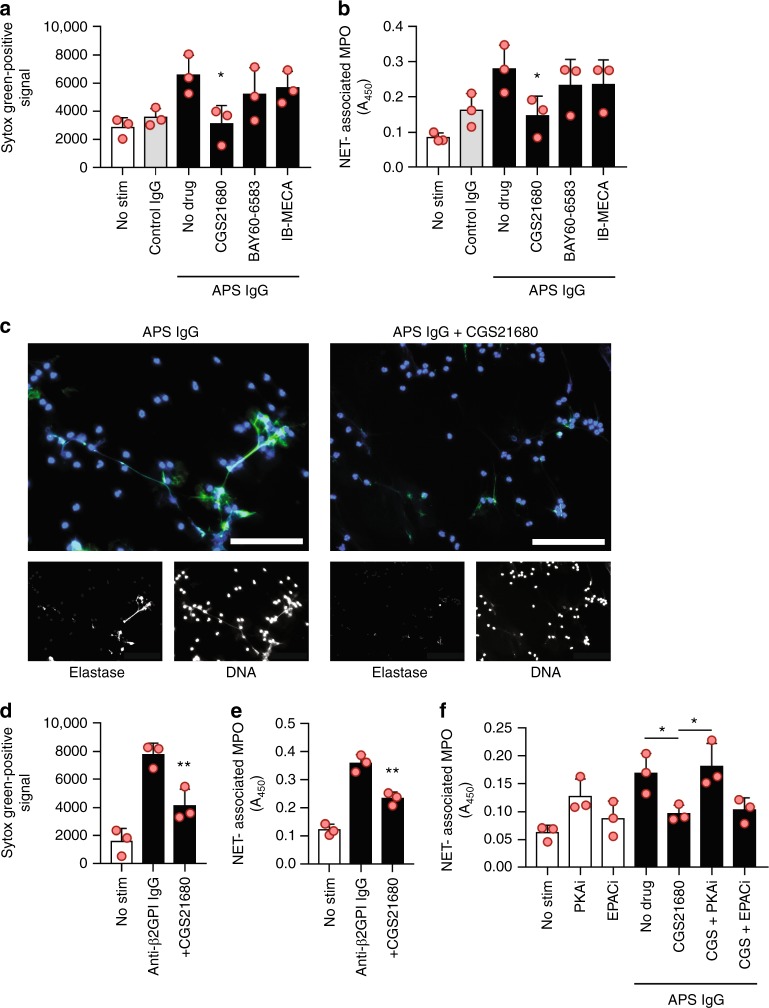
Agonism of the adenosine A_2A_ receptor suppresses NETosis. **a**, **b** Neutrophils were isolated from healthy volunteers and then treated with either control IgG or APS IgG for 3 h. Some samples were additionally treated with agonists of the adenosine A_2A_ receptor (CGS21680), A_2B_ receptor (BAY60-6583), or A_3_ receptor (IB-MECA) as indicated. In panel **a**, total extracellular DNA was measured as relative fluorescence units upon the addition of Sytox Green. In panel **b**, an independent set of experiments was performed, with NETosis quantified by measuring the enzymatic activity of nuclease-liberated myeloperoxidase (MPO). Mean and standard deviation are presented for *n* = 3 independent experiments; *p < 0.05 as compared with the APS IgG/no-drug group by one-way ANOVA corrected with Sidak’s test. **c** NETosis was assessed qualitatively by immunofluorescence microscopy. Blue = DNA, green = extracellular neutrophil elastase, and scale bar = 100 microns. **d**, **e** Neutrophils were treated with affinity-purified anti-β_2_GPI IgG (black bars) in the presence or absence of the A_2A_ receptor agonist. Extracellular DNA (**d**) or NETs (**e**) were quantified as above. Mean and standard deviation are presented for *n* = 3 independent experiments; **p < 0.01 as compared with the no-drug group by one-way ANOVA corrected with Dunnett’s test. **f** Neutrophils were treated with APS IgG (black bars) in the presence of a protein kinase A inhibitor (PKAi) or an inhibitor of the exchange protein activated by cyclic-AMP pathway (EPACi) as indicated, and NETs were quantified. Mean and standard deviation are presented for *n* = 3 independent experiments; *p < 0.05

As surface adenosine receptors are key regulators of neutrophil cAMP levels^22^, we asked whether agonism of the adenosine A_2A_, A_2B_, or A_3_ receptor might regulate NETosis. An A_2A_ agonist (CGS21680) suppressed APS IgG-mediated NETosis with nanomolar potency. In contrast, an A_2B_ agonist (BAY60-6583) and an A_**3**_ agonist (2-Cl-IB-MECA) did not modulate NETosis (Fig. 2a-c), even at concentrations as high as 10 µM (Supplementary Fig. 1). A second A_2A_ agonist (regadenoson/Lexiscan), which is approved for use in humans, was also tested, and resulted in similar suppression of NETosis (Supplementary Figure 2). Furthermore, suppression by CGS21680 was similarly effective when NETosis was activated by anti-β_2_GPI IgG that had been affinity-purified from the serum of an APS patient (Fig. 2d, e). To determine the extent to which CGS21680-mediated suppression required cAMP, we tested inhibitors of two key cAMP-dependent pathways in neutrophils: protein kinase A (PKA) and exchange protein activated by cyclic AMP (EPAC). While an EPAC inhibitor had no effect on the ability of CGS21680 to suppress APS IgG-mediated NETosis, suppression was completely neutralized by a PKA inhibitor (Fig. 2f). In summary, these data demonstrate that the adenosine A_2A_ receptor agonist CGS21680 suppresses NETosis triggered by APS patient antibodies, and that PKA is a downstream effector of this suppression.

### Adenosine A_2A_ receptor agonism suppresses ROS formation

**Fig. 3:**
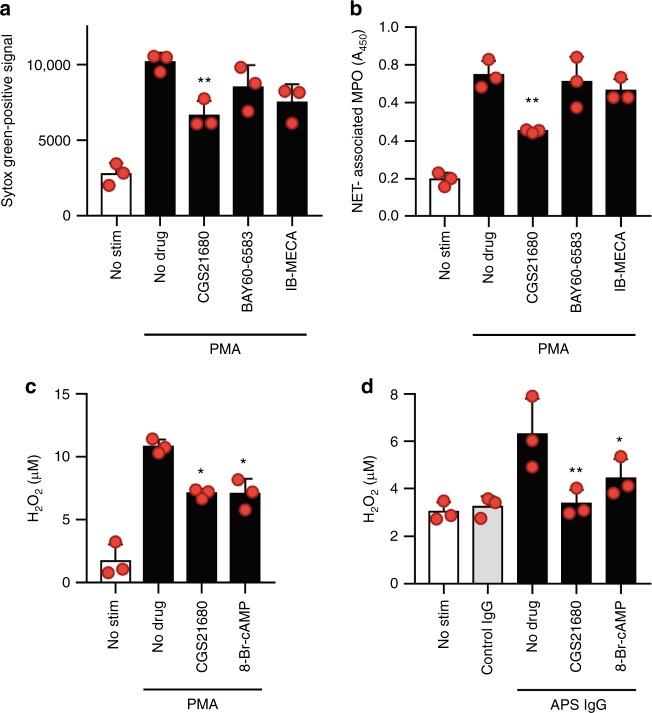
An adenosine A_2A_ receptor agonist suppresses reactive oxygen species (ROS). **a**, **b** Neutrophils were isolated from healthy volunteers and then treated with PMA for 3 h. Some samples were additionally treated with agonists of the adenosine A_2A_ receptor (CGS21680), A_2B_ receptor (BAY60-6583), or A_3_ receptor (IB-MECA). In panel **a**, total extracellular DNA was measured as relative fluorescence units upon the addition of Sytox Green. In panel **b**, an independent set of experiments was performed, with NETosis quantified by measuring the enzymatic activity of nuclease-liberated myeloperoxidase (MPO). Mean and standard deviation are presented for *n* = 3 independent experiments; **p < 0.01 as compared with the PMA/no-drug group by one-way ANOVA corrected with Dunnett’s test. **c**, **d** Neutrophils were treated with PMA, control IgG, or APS IgG in the presence or absence of the A_2A_ receptor agonist or 8-Br-cAMP for 1 h. Hydrogen peroxide formation was measured by a colorimetric assay. Mean and standard deviation are presented for *n* = 3 independent experiments; *p < 0.05 and **p < 0.01 as compared with the PMA-alone group (**c**) or APS-IgG-alone group (**d**) bye one-way ANOVA corrected with Dunnett’s test

To understand the mechanism by which adenosine A_2A_ receptor agonism mitigates NETosis, we asked whether inhibition would extend to NETosis activated by phorbol 12-myristate 13-acetate (PMA). Indeed, PMA-mediated NETosis was effectively suppressed by CGS21680 (Fig. 3a, b). PMA activates protein kinase C in neutrophils and in doing so triggers the assembly and activation of the NADPH oxidase complex, which is required for efficient NETosis in response to many stimuli^23^. As expected, PMA triggered the formation of H_2_O_2_ by neutrophils, which was suppressed by CGS21680 (Fig. 3c). These results were mirrored by APS IgG, with induction of H_2_O_2_ in neutrophils that could be suppressed by CGS21680 (Fig. 3d). In summary, these data reveal a potential mechanism by which CGS21680 suppresses NETosis, namely the suppression of NADPH oxidase-generated ROS.

### Dipyridamole suppresses NETosis **via** adenosine A_2A_ receptor

**Fig. 4:**
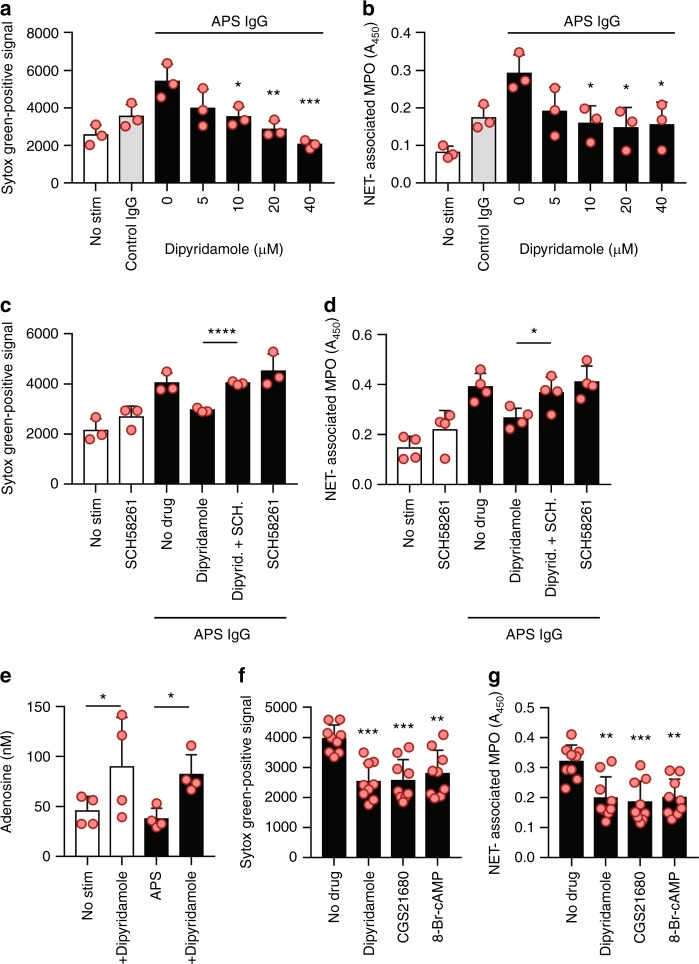
Dipyridamole suppresses NETosis via activation of the adenosine A_2A_ receptor. **a**, **b** Neutrophils were isolated from healthy volunteers and then treated with either control IgG or APS IgG for 3 h. Some samples were additionally treated with dipyridamole as indicated. In panel **a**, total extracellular DNA was measured as relative fluorescence units upon the addition of Sytox Green. In panel **b**, an independent set of experiments was performed, with NETosis quantified by measuring the enzymatic activity of nuclease-liberated myeloperoxidase (MPO). Mean and standard deviation are presented for *n* = 3 independent experiments; *p < 0.05, **p < 0.01, and ***p < 0.001 as compared with the 0 µM group by one-way ANOVA corrected with Dunnett’s test. **c**, **d** Neutrophils were treated with APS IgG in the presence of different combinations of dipyridamole and the A_2A_ receptor antagonist SCH58261. Extracellular DNA (**c**) or NETs (**d**) were quantified as above. Mean and standard deviation are presented for *n* = 3 independent experiments; *p < 0.05 and **p < 0.01 by one-way ANOVA. **e** Neutrophils were treated with APS IgG (black bars) and dipyridamole as indicated. Adenosine was quantified in culture supernatants. Mean and standard deviation are presented for *n* = 4 independent experiments; *p < 0.05 by one-way ANOVA corrected with Sidak’s test. **f**, **g** Neutrophils were isolated from patients with primary APS and immediately placed in culture. Some samples were additionally treated with drugs as indicated. Extracellular DNA (**f**) or NETs (**g**) were quantified as above. Mean and standard deviation are presented for *n* = 9 patients; **p < 0.01 and ***p < 0.001 as compared with the no-drug group by one-way ANOVA corrected with Dunnett’s test

The antithrombotic medication dipyridamole is known to potentiate adenosine receptor-mediated signaling by (i) increasing extracellular concentrations of adenosine, and (ii) interfering with the breakdown of cAMP^24^. Indeed, dipyridamole suppressed APS IgG-mediated NETosis (Fig. 4a, b), an effect which could be fully reversed with an A_2A_ receptor antagonist (Fig. 4c, d). Consistent with its role as an antagonist of adenosine uptake into cells, we demonstrated that dipyridamole raised adenosine levels in culture supernatants (Fig. 4e). Beyond in vitro-activated neutrophils, we also found that dipyridamole suppressed the spontaneous NETosis of neutrophils freshly isolated from primary APS patients (Fig. 4f, g). Similar suppression was seen with CGS21680 and 8-Br-cAMP (Fig. 4f, g). The clinical characteristics of these patients are described in Supplementary Table 1. In summary, these data demonstrate that dipyridamole phenocopies CGS21680 by suppressing NET release from APS neutrophils via activation of the adenosine A_2A_ receptor.

### Adenosine A_2A_ receptor agonism mitigates venous thrombosis

**Fig. 5:**
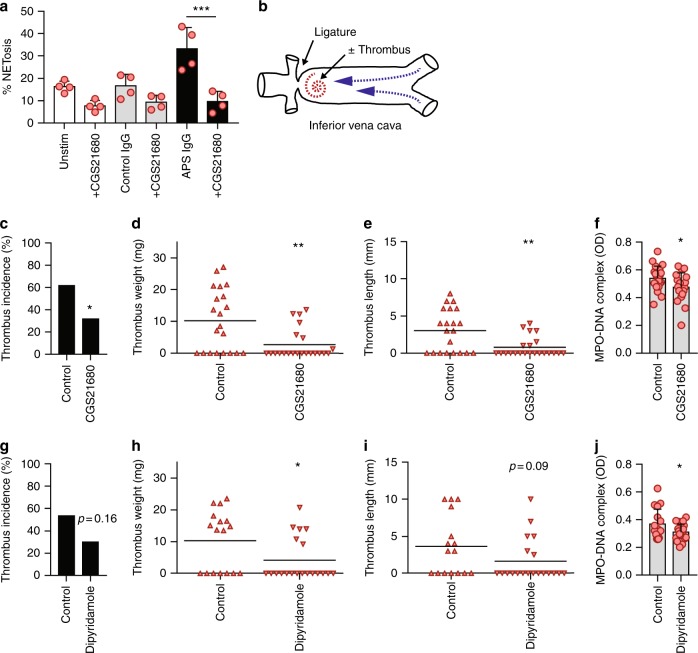
Agonism of the adenosine A_2A_ receptor mitigates venous thrombosis in control mice. **a** Neutrophils were prepared from the bone marrow of C57BL/6 mice and then treated with control IgG or APS IgG. Some samples were additionally treated with CGS21680. NETosis was quantified by immunofluorescence microscopy. Mean and standard deviation are presented for *n* = 3 independent experiments; ***p < 0.001 by one-way ANOVA corrected with Sidak’s test. **b** Schematic of the flow-restriction model of venous thrombosis. The inferior vena cava is narrowed by fixing a ligature around the IVC (using a spacer, which is then removed). When a thrombus forms, it is just caudal to the stenosis in the area indicated by the spiral. Thrombus formation was assessed at 48 h for all of the following experiments. **c**–**e** Thrombus incidence (**c**), thrombus weight (**d**), and thrombus length (**e**) were assessed for C57BL/6 wild-type mice subjected to the flow-restriction model. Some mice were treated with the adenosine A_2A_ receptor agonist CGS21680 as indicated. Each data point represents a unique mouse, while horizontal lines denote mean values; *p < 0.05 and **p < 0.01 as compared with the control group (Chi-square test for panel **c** and unpaired *t-*test for panels **d** and **e**). **f** Plasma NETs were measured by myeloperoxidase (MPO)-DNA ELISA 48 h after flow restriction; *p < 0.05 as compared with the control group. **g**–**i** Thrombus incidence (**g**), thrombus weight (**h**), and thrombus length (**i**) were assessed for mice subjected to the flow-restriction model. Some mice were treated with dipyridamole as indicated; *p < 0.05 as compared with the control group (Chi-square test for panel **g** and unpaired *t-*test for panels **h** and **i**). **j** Plasma NETs were measured by myeloperoxidase (MPO)-DNA ELISA 48 h after flow restriction; *p < 0.05 as compared with the control group by unpaired *t-*test

Studies in humans have implicated neutrophils and NETs in pathologic thrombosis across various vascular beds^25–27^. At the same time, our group and others have proven a role for NETs in mouse models of venous thrombosis^12,28,29^. Here, we reasoned that CGS21680, which suppresses NETosis by human neutrophils in vitro, might also mitigate venous thrombosis in vivo. We first demonstrated that CGS21680 reduced NETosis in murine neutrophils in vitro (Fig. 5a), similar to what we had observed in human neutrophils (Fig. 2a-c). Then, in a flow-restriction model of inferior vena cava (IVC) thrombosis (Fig. 5b), we found that administration of CGS21680 not only reduced thrombus incidence, weight, and length (Fig. 5c-e), but also lowered plasma NET levels (Fig. 5f). Administration of CGS21680 did not affect the blood pressure or heart rate of treated mice (Supplementary Figure 3). Interestingly, CGS21680 was not effective in a different model of venous thrombosis (full-ligation/stasis) that demonstrates less neutrophil dependence (Supplementary Fig. 4)^30^. In summary, these data are the first to demonstrate the efficacy of an adenosine A_2A_ receptor agonist against venous thrombosis.

### Dipyridamole suppresses NETosis and venous thrombosis

We next asked whether dipyridamole would, similar to CGS21680, mitigate venous thrombosis and the formation of plasma NETs in the context of the flow-restriction model. Indeed, dipyridamole reduced thrombus weight, albeit without a statistically significant effect on incidence or length (Fig. 5g-i). Plasma NET levels were also significantly decreased when mice subjected to the flow-restriction model were treated with dipyridamole (Fig. 5j). In summary, dipyridamole suppresses NETosis and venous thrombosis in vivo.

### Adenosine A_2A_ receptor agonism mitigates APS thrombosis

**Fig. 6:**
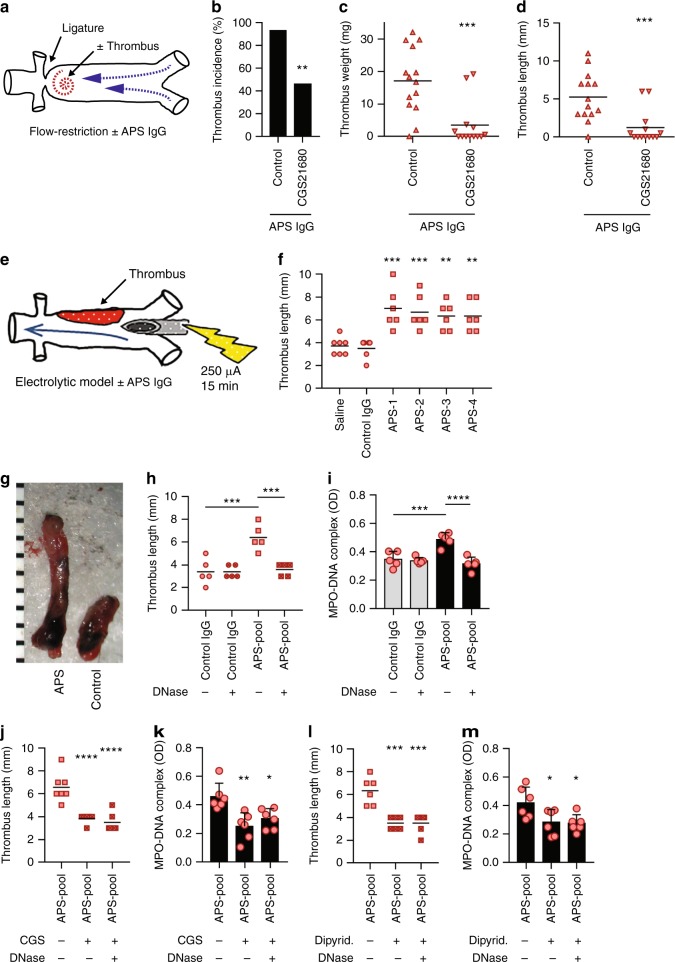
Agonism of the adenosine A_2A_ receptor mitigates venous thrombosis in APS mice. **a**–**d** Thrombus incidence (**b**), thrombus weight (**c**), and thrombus length (**d**) were assessed for mice subjected to the flow-restriction model (**a**). Here, mice were additionally treated with APS IgG as described in Methods. Each data point represents a unique mouse, while horizontal lines denote mean values; **p < 0.01 and ***p < 0.001 as compared with the control group (Chi-square test for panel **b** and unpaired *t-*test for panels **c**, **d**). **e** Schematic of the electrolytic model of venous thrombosis. Direct current results in the release of free radicals within the IVC, which activate endothelial cells and initiate a thrombogenic environment in the presence of constant blood flow. Thrombus formation was assessed at 24 h for all of the following experiments. **f**, **g** Thrombus size (**f**) and representative thrombi (**g**) for mice treated with control IgG or APS IgG as indicated. In this experiment, APS-1 through −4 represent unique patients. Each data point represents a unique mouse, while horizontal lines denote mean values; **p < 0.01 and ***p < 0.001 as compared with the control group by one-way ANOVA corrected with Dunnett’s test. The ruler denotes millimeters. **h**, **i** Thrombus size and plasma MPO-DNA complexes were determined for mice treated with various combinations of control IgG, APS IgG, and intravenous deoxyribonuclease (DNase) as indicated; ***p < 0.001 and ****p < 0.0001 as indicated by one-way ANOVA corrected with Sidak’s test (**h**) or Dunnett’s test (**i**). **j**, **k** Thrombus size and plasma MPO-DNA complexes were determined for mice treated with various combinations of APS IgG, CGS21680, and DNase as indicated; *p < 0.05, **p < 0.01, and ****p < 0.0001 as compared with the control group by one-way ANOVA corrected with Dunnett’s test. **l**, **m** Thrombus size and plasma MPO-DNA complexes were determined for mice treated with various combinations of APS IgG, dipyridamole, and DNase as indicated; *p < 0.05 and ***p < 0.001 as compared with the control group by one-way ANOVA corrected with Dunnett’s test

In the flow-restriction model, we have previously demonstrated that the administration of IgG isolated from APS patients potentates thrombosis^12^. Here, we found that CGS21680 is highly effective in this APS model, reducing thrombus incidence, weight, and length (Fig. 6a-d). We next introduced a second model of IVC thrombosis to further examine APS-accelerated thrombosis in the context of active blood flow. In the electrolytic model (Fig. 6e), the application of mild direct electrical current results in the release of free radicals within the IVC, which activate endothelial cells and initiate a thrombogenic environment in the presence of constant blood flow^31^. First we revealed that administration of APS IgG (isolated from individual patients), but not control IgG, potentiated thrombus size (Fig. 6f, g). Importantly, both thrombus size and NET release were reduced by treatment with intravenous deoxyribonuclease (DNase) (Fig. 6h, i). Going further, we found that both CGS21680 (Fig. 6j, k) and dipyridamole (Fig. 6l, m) functioned similarly to DNase, with no synergy appreciated between DNase and the A_2A_ receptor agonists. In summary, these data are the first to demonstrate the efficacy of adenosine A_2A_ receptor agonists against APS-accelerated venous thrombosis, with agonists functioning almost identically to DNase administration in the electrolytic model.

## Discussion

APS is an autoimmune disorder that is primarily treated with anticoagulant, rather than immunomodulatory, medications. While anticoagulation is somewhat effective against the thrombotic and pregnancy complications of APS, the approach comes with inherent and sometimes life-threatening complications, especially bleeding. Furthermore, anticoagulation has little proven efficacy in other APS manifestations such as cardiac valve disease, thrombocytopenia, nephropathy, cognitive decline, and seizures. While current clinical efforts are primarily focused on testing direct oral anticoagulants in APS patients^32^, we would argue that better understanding the inflammatory pathophysiology of APS must continue to be emphasized, with the goal of establishing more targeted and effective therapeutics. Here, we demonstrate that a specific adenosine A_2A_ receptor agonist can attenuate APS IgG-mediated NETosis in vitro and venous thrombosis in mice. Furthermore, dipyridamole (an FDA-approved drug known to potentiate adenosine signaling) phenocopies adenosine A_2A_ receptor agonism in terms of suppressing both NETosis and venous thrombosis.

Despite the abundance of neutrophils in peripheral blood (and their increasingly recognized role in pathological thrombosis), three other cell types—monocytes, endothelial cells, and platelets—have historically received more attention as drivers of thrombosis in APS^33^. This work has demonstrated that antiphospholipid antibodies actually engage phospholipid-binding proteins such as β_2_GPI, which are found in association with cell-surface phospholipids^34^. These interactions lead to cell activation and upregulation of prothrombotic molecules, including tissue factor, a potent mediator of thrombosis^35^. More recently, our group and others have suggested a role for neutrophils in the prothrombotic diathesis inherent to APS^11–13,36–40^. These studies were triggered by an emerging emphasis in the thrombosis literature regarding the importance of neutrophils, their heterotypic cell–cell interactions, and NETs in pathologic clotting—and, in particular, venous thrombosis^26^. As examples, histones stimulate platelets;^41^ the DNA component of NETs activates the intrinsic coagulation cascade;^28,42^ and NET-derived proteases inactivate certain anticoagulant factors^28^. In some contexts, NETs may also be an important source of tissue factor^43^.

Our recent transcriptomic profiling of APS neutrophils revealed an activated signature with upregulation of complement and Fc receptors, selectins and their ligands, and interferon-responsive pathways, among other genes^13^. Pathway analysis demonstrated upregulation of three subunits of PKA in a node associated with metabolic processes: specifically two PKA catalytic subunits (PRKACA and PRKACG) and one PKA regulatory subunit (PRKAR1A)^13^. This upregulation would seem to support PKA activation as a potential therapeutic strategy in APS (for example, with drugs such as phosphodiesterase 4 inhibitors)^44^. Regarding mechanism, the PKA pathway has been shown to downregulate the phosphorylation of the p47 subunit of NADPH oxidase, which prevents its translocation to the membrane for assembly and activation with other subunits^45^. Indeed, it is interesting that inhibition of PKA seems to activate NETosis in resting control neutrophils (Fig. 2f). Further studies should explore the extent to which cAMP/PKA functions as a homeostatic pathway that keeps NETosis at bay, perhaps especially in adherent neutrophils. It may also be interesting to determine the extent to which the cAMP/PKA axis can counterbalance signaling through surface receptors such as the C5a receptor, which our previous work has revealed to be upregulated in APS neutrophils^13^.

For in vivo studies, it is possible (if not likely) that agonism of the adenosine A_2A_ receptor has antithrombotic properties that extend beyond suppression of NETosis—properties which may take on increased importance depending on which vascular bed is being studied. For example, beyond neutrophils, platelets express the A_2A_ receptor, which suppresses platelet aggregation when activated^46^, an effect that may be especially relevant in the context of arterial thrombosis. In the data presented here, we find that both CGS21680 and dipyridamole are highly effective against APS IgG-accelerated venous thrombosis, reducing thrombus size to the levels seen in control mice (these data are essentially indistinguishable from what we see when mice are treated with systemic DNase). Given that all three treatments (CGS21680, dipyridamole, and DNase) reduce plasma levels of NETs in similar fashion, and given that we do not see synergy when combining treatments (for example, CGS21680 and DNase), we suspect that both CGS21680 and dipyridamole are acting primarily through neutrophil inhibition in these models. Going forward, this question may be additionally interrogated via the development of transgenic mice lacking the A_2A_ receptor. Such mice could be used to further define thrombosis in large veins, while also asking questions about the role the neutrophil A_2A_ receptor plays in other vascular beds. Indeed, our group and others have proposed that different cell types likely take on a different level of importance in APS depending on the vascular bed in question. As such, a drug with pleomorphic antithrombotic effects such as dipyridamole might be especially attractive for use in such patients^47^.

All mouse studies were performed in male mice given the more consistent surgical result in our hands (which we attribute to more consistent IVC anatomy). While many APS cohorts include more females than males (given associations of APS with pregnancy morbidity and female-predominant lupus)^2^, the sex distribution is relatively equal between females and males when one considers primary thrombotic APS^48^—which we consider the clinical framework for the data presented here. Of course, one might ask whether A_2A_ receptor agonism would benefit other manifestations of APS beyond thrombosis such as pregnancy loss. While dipyridamole has a relatively robust safety record in pregnancy^49^, we would consider such discussions premature, especially given that obstetric APS may have very different pathophysiology from thrombotic APS^50^.

Dipyridamole is known to potentiate adenosine receptor-mediated signaling by both increasing extracellular concentrations of adenosine and inhibiting phosphodiesterases that degrade cAMP^24^. Unlike regadenoson (Supplementary Fig. 2), which has a short half-life in vivo and would require administration by infusion^51,52^, dipyridamole has a profile compatible with long-term administration^53^. While dipyridamole has never been systematically tested in patients with APS, drugs with similar adenosine-amplifying properties, such as dilazep^54^ and defibrotide^55^, have been reported as effective in either case reports or preclinical models. The data presented here suggest the potential for an anti-inflammatory therapeutic paradigm in APS, which may even extend to selected cases of venous thrombotic disease in the general population.

## Methods

### Purification of patient IgG

This study complied with all relevant ethical regulations and was approved by the University of Michigan Institutional Review Board; all participants provided informed consent for blood donation. IgG was purified from APS or control sera with a Protein G Agarose Kit following the manufacturer’s instructions (Pierce). Briefly, serum was diluted in IgG binding buffer and passed through a Protein G Agarose column at least five times. IgG was then eluted with 0.1 M glycine and then neutralized with 1 M Tris. This was followed by overnight dialysis against PBS at 4 °C. IgG purity was verified with Coomassie staining, and concentrations were determined by BCA protein assay (Pierce) according to manufacturer’s instructions. All IgG samples were determined to be free of detectable endotoxin by the Pierce LAL Chromogenic Endotoxin Quantitation Kit (88282) according to manufacturer’s instructions.

### Human neutrophil purification and NETosis assays

For neutrophil preparation, blood from either healthy volunteers or APS patients was collected into heparin tubes by standard phlebotomy techniques. The anticoagulated blood was then fractionated by density-gradient centrifugation using Ficoll-Paque Plus (GE Healthcare). Neutrophils were further purified by dextran sedimentation of the red blood cell layer, before lysing residual red blood cells with 0.2% sodium chloride. Neutrophil preparations were at least 95% pure as confirmed by both flow cytometry and nuclear morphology.

To assess NETosis, complementary approaches were utilized. For the SYTOX Green assay, neutrophils were resuspended in RPMI media (Gibco) supplemented with 0.5% bovine serum albumin (BSA, Sigma) and 0.5% fetal bovine serum (Gibco), which had been heat-inactivated at 56 °C. Neutrophils (1 × 10^5^/well) were then incubated in 96-well black microplates (with clear flat bottoms) at 37 ^°^C with 100 nM PMA (Sigma), 10 µg ml^−1^ APS IgG, or 100 ng ml^−1^ anti-β_2_GPI IgG in the presence or absence of different agonists/inhibitors, including 8-Br-cAMP (cAMP mimetic), forskolin (adenylyl cyclase activator, 20 µM), Ro 20-1724 (phosphodiesterase 4 inhibitor 10 µM), CGS21680 (A_2A_ agonist, 100 nM), BAY60-6583 (A_2B_ agonist, 100 nM), IB-MECA (A_3_ agonist, 100 nM), regadenoson (Lexiscan, 100 nM), KT5720 (PKA inhibitor, 10 µM), ESI09 (EPAC inhibitor, 10 µM), dipyridamole (10 µM), and SCH58261 (A_2A_ antagonist, 10 µM). All these chemicals were from Tocris, except for dipyridamole (Sigma) and regadenoson (University of Michigan hospital pharmacy). After 3 h, SYTOX Green (Thermo Fisher Scientific) was added to a final concentration of 0.2 µM and incubated for an additional 10 min. Fluorescence was quantified at excitation and emission wavelengths of 485 nm and 520 nm, respectively, using a Cytation 5 Cell Imaging Multi-Mode Reader (BioTek) with the following settings. Light Source: Xenon Flash. Lamp Energy: High. Extended Dynamic Range Read Speed: Normal. Delay: 10 msec. Measurements/Data Point: 10. Read Height: 7 mm. On each day of the experiment, wells were set up in triplicate and the results averaged to obtain a single data point. All experiments were performed at least three times (i.e., independent experiments on three separate days).

For the NET-associated myeloperoxidase (MPO) assay, neutrophils were cultured as above in 96-well plates. To collect NET-associated MPO, the culture media was discarded (to remove any soluble MPO) and replaced with 100 µL of RPMI supplemented with 5 U ml^−1^ Micrococcal nuclease (Thermo Fischer Scientific). After 10 min at 37 ^°^C, digestion of NETs was stopped with 10 mM EDTA. Supernatants were transferred to a v-shaped 96-well plate, and centrifuged at 350 × *g* for 5 min to remove debris. Supernatants were then transferred into a new plate. To measure for MPO activity, an equal volume of 3,3’,5,5’-Tetramethylbenzidine (TMB) substrate (1 mg ml^−1^, Thermo Fischer Scientific) was added to each well. After 10 min of incubation in the dark, the reaction was stopped by the addition of 50 µL of 1 mM sulfuric acid. Absorbance was measured at 450 nm using a Cytation 5 Cell Imaging Multi-Mode Reader.

For immunofluorescence microscopy, 1.5 × 10^5^ neutrophils were seeded onto coverslips coated with 0.001% poly-L-lysine (Sigma) and fixed with 4% paraformaldehyde. In some experiments, cells were then permeabilized with 0.1% Triton-X for 15 min at room temperature. Blocking was with 1% bovine serum albumin. The primary antibody was against neutrophil elastase (Abcam 21595, diluted 1:100), and the FITC-conjugated secondary antibody was from SouthernBiotech (4052-02, diluted 1:250). DNA was stained with Hoechst 33342 (Invitrogen). Images were collected with a Cytation 5 Cell Imaging Multi-Mode Reader.

### Purification of β_2_GPI and IgG anti-β_2_GPI

β_2_GPI was isolated from pooled human serum (Innovative Research) as describe previously^56^, with slight modifications. Briefly, 150 ml of human serum was diluted with an equal volume of normal saline, followed by treatment with 6% perchloric acid (Sigma) at 4 ^°^C to a final concentration of 0.285 M. The mixture was stirred for 15 min at 4 ^°^C, and the supernatant was collected by centrifugation at 10,000 × *g* for 15 min at 4 °C. The supernatant pH was adjusted to 8.0 and was dialyzed against 0.02 M NaCl + 0.03 M Tris pH 8.0 overnight at 4 °C. β_2_GPI was then purified using a HiTrap Heparin column (GE Healthcare) in an AKTA Prime chromatographic system (GE Healthcare) at 4 °C. The adsorbed protein was eluted stepwise with 0.02 M Tris buffer pH 8.0 with increasing molarities of NaCl (0.03, 0.15, and 0.35 M). The fractions eluted with 0.035 M NaCl were pooled and dialyzed overnight against 0.05 M sodium acetate. These fractions were then further purified using Resource S columns (GE Healthcare). Elutes were collected using a 0–100% gradient from 0.05 M sodium acetate pH 4.8 + 0.05 M NaCl to 0.05 M sodium acetate pH 5.2 + 0.65 M NaCl. Protein fractions were identified by SDS PAGE, pooled, and dialyzed overnight against PBS at 4 °C.

For preparation of anti-β_2_GPI IgG, total IgG was first prepared from patient serum using protein G agarose columns as described previously^11^. β_2_GPI protein was coupled to an N-hydroxysuccinimide-activated HiTrap column (GE Healthcare) according to the manufacturer’s instructions. IgG anti-B2GPI was then purified using an approach similar to what has been described previously^57^. All steps were performed at room temperature. Briefly, total IgG was diluted 1:1 with binding buffer (0.01 M sodium phosphate pH 7.0). The column was then primed with 5 column volumes of binding buffer, 5 column volumes of elution buffer (0.1 M glycine-HCl, pH 2.7), and finally another 5 column volumes of binding buffer. The IgG solution was then passed through the column twice. After washing with 5 column volumes of binding buffer, the bound IgG was eluted with elution buffer. Eluted fractions were immediately neutralized with 1 M Tris-HCl pH 9.0. Eluents with high protein content were pooled and dialyzed against PBS overnight at 4 °C.

### H_2_O_2_ assay

The generation of H_2_O_2_ was quantified, essentially as described previously^58^. Briefly, H_2_O_2_ production was detected by a colorimetric assay, with 50 µM Amplex Red reagent (Invitrogen) and 10 U ml^−1^ horseradish peroxidase (Sigma) added to the culture medium. Absorbance was measured at 560 nm and linearity was assured with an H_2_O_2_ standard curve.

### Animal housing and treatments

Mice were housed in a specific pathogen-free barrier facility, and fed standard chow. Experimental protocols were approved by the University of Michigan Institutional Animal Care and Use Committee, and all relevant ethical regulations were followed. Male C57BL/6 mice were purchased from The Jackson Laboratory. CGS21680 (Tocris Bioscience) was administered by intraperitoneal injection, 0.5 mg kg^−1^ twice daily. Pharmaceutical-grade dipyridamole (NDC 0641-2569-44) was administered by intraperitoneal injection (5 mg kg^−1^). Some mice were additionally administered DNase (Pulmozyme/dornase alfa, Genentech) every 12 h: 50 μg by intraperitoneal injection and 10 μg by intravenous (retro-orbital) injection with each dose. Therapy was always started one day before IVC surgery, and continued through the duration of the experiment.

### Mouse neutrophil purification and NETosis assay

Bone marrow neutrophils were isolated according to our usual protocol^58,59^. Total bone marrow cells were spun on a discontinuous Percoll gradient (52%-69%-78%) at 1500 × *g* for 30 min. Cells from the 69–78% interface were collected. These cells were >95% Ly-6G-positive by flow cytometry, and had typical nuclear morphology by microscopy. To assess in vitro NETosis, a protocol similar to what we have described previously was followed^58,59^. Culture was for 4 h at 37 °C in RPMI media supplemented with 2% bovine serum albumin and 10 mM HEPES buffer. Stimulation was with control IgG or APS IgG at a concentration of 10 µg ml^−1^. For immunofluorescence, cells were fixed with 4% paraformaldehyde. DNA was stained with Hoechst 33342 (Invitrogen), while protein staining was with rabbit polyclonal antibody to citrullinated histone H3 (Abcam 5103, diluted 1:100), followed by FITC-conjugated anti-rabbit IgG (SouthernBiotech 4052-02, diluted 1:250). Images were collected with a Cytation 5 Cell Imaging Multi-Mode Reader. NETs (decondensed extracellular DNA co-staining with citrullinated histone H3) were quantified by two blinded observers, and digitally recorded to prevent multiple counts. The percentage of NETs was calculated after counting 10 200x fields per sample^12^.

### **In vivo** venous thrombosis

To model large-vein thrombosis, we employed procedures that we have utilized previously^12,13^. For the stenosis model, male C57BL/6 wild-type mice were administered two doses (500 µg each) of either control or APS IgG by intraperitoneal injection, 48 h apart. Just prior to the second injection, a laparotomy was performed under anesthesia, and a ligature was fastened around the IVC over a blunted 30-gauge needle (which served as a spacer); side and back branches were not manipulated. After removal of the spacer, the abdomen was closed, and the mouse was allowed to recover. In separate experiments to examine complete stasis, the IVC was fully ligated, without use of a spacer, and all visible side and back branches were also ligated^12^. Mice were humanely euthanized and thrombus formation was assessed 48 h after laparotomy.

The electrolytic model was performed as described^31^. Briefly, after exposure of the IVC, any lateral branches were ligated using 7‐0 Prolene suture (back branches remained patent). A 30‐gauge silver-coated copper wire (KY‐30‐1‐GRN, Electrospec) with exposed copper wire at the end was placed inside a 25‐gauge needle, which was inserted into the IVC and positioned against the anterior wall (where it functioned as the anode). Another needle was implanted subcutaneously, completing the circuit (cathode). A constant current of 250 μA was applied for 15 min. The current was supplied by the voltage‐to‐current converter that is described in detail in the ref. ^31^. After removal of the needle, the abdomen was closed. Before recovery from anesthesia, mice received a single intravenous injection of either control or APS IgG (500 µg). Twenty-four hour later, mice were humanely euthanized, blood was collected, and thrombus length was measured.

### Quantification of MPO-DNA complexes

MPO-DNA complexes were quantified similarly to what has been previously described^60^. This protocol used several reagents from the Cell Death Detection ELISA kit (Roche). First, a high-binding EIA/RIA 96-well plate (Costar) was coated overnight at 4 °C with anti-human MPO antibody (Bio-Rad 0400-0002), diluted to a concentration of 2 µg ml^−1^ in coating buffer (Cell Death kit). The plate was washed three times with wash buffer (0.05% Tween 20 in PBS), and then blocked with 1% bovine serum albumin in PBS for 90 min at room temperature. The plate was again washed three times, before incubating for 1 h at room temperature with 10% serum or plasma in the aforementioned blocking buffer. The plate was washed five times, and then incubated for 90 min at room temperature with 1x anti-DNA antibody (HRP-conjugated; Cell Death kit) diluted 1:20 in blocking buffer. After five more washes, the plate was developed with 3,3’,5,5’-Tetramethylbenzidine (TMB) substrate (Invitrogen) followed by a 2 N sulfuric acid stop solution. Absorbance was measured at a wavelength of 450 nm with a Synergy HT Multi-Mode Microplate Reader (BioTek). Data were normalized to an in vitro-prepared NET standard, included on every plate.

### Blood pressure

Non-invasive blood pressure was measured by tail cuff as described^61^. Briefly, using the IITC Life Science blood pressure measurement system, conscious and restrained mice were acclimated for 3 days in a temperature controlled environment (model 306 warming chamber). The tail vein was occluded with an integrated sensor-cuff (model I-B60-1/4) and return of pulsation (RTP) detected by the RTP-computerized blood pressure monitor (model 6 M 229 6 channel mouse system). Repeated measures were averaged for determination and report of systolic blood pressure and heart rate.

### Measurement of adenosine levels in culture supernatants

Neutrophils (2 × 10^6^/ml) were incubated in 12-well plates at 37 ^°^C with 10 µg ml^−1^ APS IgG in the presence or absence of dipyridamole (10 µM). After 3 h, 1-ml supernatants were collected and centrifuged at 9000 × *g* for 5 min to remove insoluble particles. Supernatants were assayed without dilution via the Adenosine Assay Kit MET-5090 (Cell Biolabs, Inc) according to the manufacturer’s instructions.

### Statistical analysis

Data analysis was with GraphPad Prism software version 8. For continuous variables, group means were compared by either *t*-test (two groups) or one-way ANOVA (more than two groups); correction for multiple comparisons was by Dunn’s method. For categorical variables, analysis was by Chi-square test. Statistical significance was defined as *p* < 0.05.

### Reporting summary

Further information on research design is available in the Nature Research Reporting Summary linked to this article.

## Supplementary information

Supplementary Information

Reporting Summary

## Data Availability

All data generated or analyzed during this study are included in the published article (and its supplementary information files). All data are available from the authors upon reasonable request.
