# Supplementary material for: Adenosine receptor agonism protects against NETosis and thrombosis in antiphospholipid syndrome
Source: Nat Commun. 2019 Apr 23;10:1916. doi: 10.1038/s41467-019-09801-x (PMC6478874; doi:10.1038/s41467-019-09801-x)
Supplement: Supplementary file 1 — Supplementary Information [file 41467_2019_9801_MOESM1_ESM.pdf]

SUPPLEMENTARY INFORMATION

Adenosine receptor agonism protects against NETosis and thrombosis in antiphospholipid syndrome

Ali *et al.*

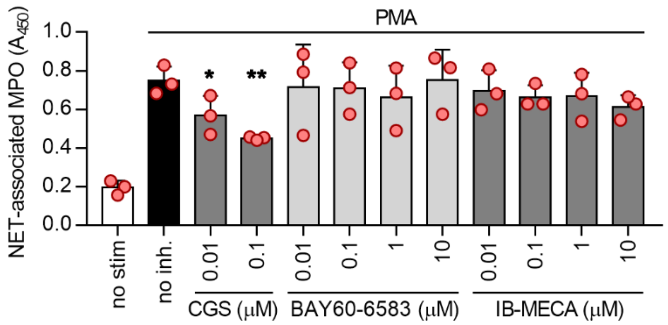

Supplementary Figure 1 Agonism of the adenosine A_{2A} receptor suppresses NETosis. **A-B,** Neutrophils were isolated from healthy volunteers and then treated with 100nM phorbol-12-myristate-13-acetate (PMA) as indicated for 3 hours. Some samples were additionally treated with agonists of the adenosine A_{2A} receptor (CGS21680), A_{2B} receptor (BAY60-6583), or A_3 receptor (IB-MECA) as indicated. NETosis was quantified by measuring the enzymatic activity of nuclease-liberated myeloperoxidase (MPO). Mean and standard deviation are presented for n=3 independent experiments; *p<0.05 and **p<0.01 as compared with the no-inhibitor condition by one-way ANOVA corrected with Dunnett's test.

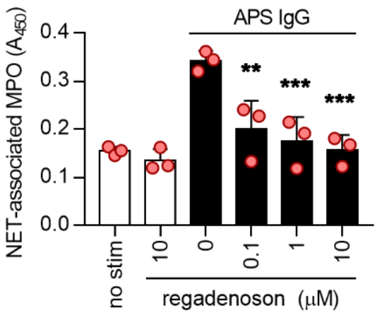

Supplementary Figure 2 Regadenoson (Lexiscan) inhibits APS IgG-mediated NETosis. Neutrophils were isolated from healthy volunteers and then treated with APS IgG and various concentrations of regadenoson for 3 hours. NETosis was quantified by measuring the enzymatic activity of nuclease-liberated myeloperoxidase (MPO). Mean and standard deviation are presented for n=3 independent experiments; **$p<0.01$ and ***$p<0.001$ as compared with the no-regadenoson group by one-way ANOVA corrected with Dunnett's test.

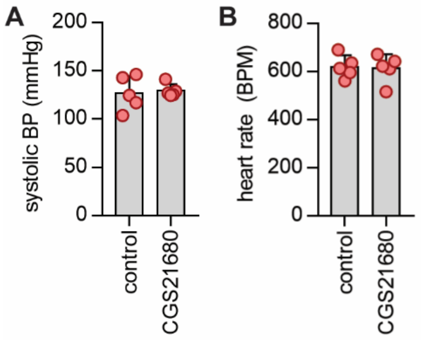

Supplementary Figure 3 Agonism of the adenosine A_{2A} receptor does not cause hypotension. C57BL/6 wild-type mice were treated with either CGS21680 or vehicle, using the same dosing scheme employed in flow-restriction experiments. **A-B,** systolic blood pressure (A) and heart rate (B) were determined as described in Methods. Mean and standard deviation are presented for n=5 mice; means are not significantly different by unpaired t test.

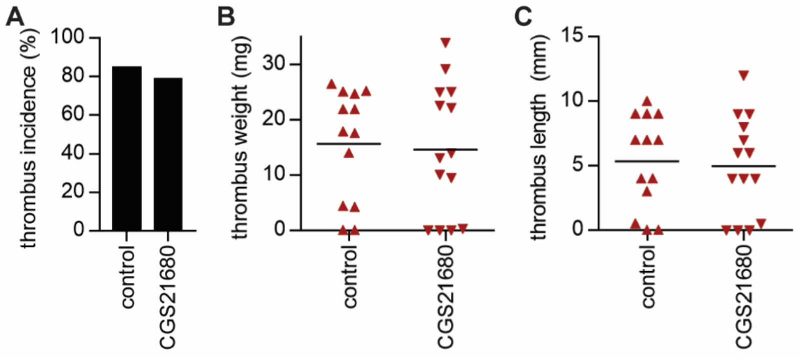

Supplementary Figure 4 Agonism of the adenosine A_{2A} receptor does not mitigate thrombosis in the stasis model. Complete ligation of the IVC and side branches was performed as described in Methods. C57BL/6 wild-type mice were treated with either CGS21680 or vehicle, using the same dosing scheme employed in flow-restriction experiments. **A-C,** Thrombus incidence (A), thrombus weight (B), and thrombus length (C) were assessed for mice subjected to the full-ligation/stasis model. Each data point represents a unique mouse, while horizontal lines denote mean values; no comparisons were statistically significant (Chi-square test for panel A and unpaired t test for panels B and C).

Supplementary Table 1 **Primary APS patients**

**Demographics**	**Neutrophils**		**IgG**	
Number of patients	9		4	
Age (y)*	44	±19	55	±16
Disease duration (y)*	12	±7	21	±8
Female gender	5	(56%)	2	(50%)
**Laboratory studies**				
IgG anti-β_2GPI	7	(78%)	4	(100%)
IgM anti-β_2GPI	3	(33%)	0	(0%)
IgA anti-β_2GPI	4	(44%)	2	(50%)
IgG anticardiolipin	7	(78%)	4	(100%)
IgM anticardiolipin	4	(44%)	0	(0%)
Lupus anticoagulant	7	(78%)	4	(100%)
Triple-positive	7	(78%)	4	(100%)
**Clinical history**				
Venous thrombosis	4	(44%)	3	(75%)
Arterial thrombosis	4	(44%)	3	(75%)
Microvascular	1	(11%)	1	(25%)
Pregnancy morbidity	2	(40%)	0	(0%)
Thrombocytopenia	4	(44%)	0	(0%)
Livedo	2	(22%)	0	(0%)
**Medications**				
Warfarin	3	(33%)	3	(75%)
Anti-platelet	6	(67%)	2	(50%)
LMWH	1	(11%)	0	(0%)
Direct oral anticoagulants	0	(0%)	0	(0%)
Hydroxychloroquine	7	(78%)	1	(25%)
Immunosuppressants	2	(22%)	1	(25%)

***** mean ± standard deviation
LMWH=low molecular weight heparin
