# Supplementary material for: Adenosine receptor agonism protects against NETosis and thrombosis in antiphospholipid syndrome
Source: Nat Commun. 2019 Apr 23;10:1916. doi: 10.1038/s41467-019-09801-x (PMC6478874; doi:10.1038/s41467-019-09801-x)
Supplement: Supplementary file 2 — Reporting Summary [file 41467_2019_9801_MOESM2_ESM.pdf]

nature research

Corresponding author(s): Jason S. Knight

Last updated by author(s): March 29, 2019

Reporting Summary

Nature Research wishes to improve the reproducibility of the work that we publish. This form provides structure for consistency and transparency in reporting. For further information on Nature Research policies, see Authors & Referees and the Editorial Policy Checklist.

Statistics

For all statistical analyses, confirm that the following items are present in the figure legend, table legend, main text, or Methods section.

n/a	Confirmed	
☐	☒	The exact sample size (*n*) for each experimental group/condition, given as a discrete number and unit of measurement
☐	☒	A statement on whether measurements were taken from distinct samples or whether the same sample was measured repeatedly
☐	☒	The statistical test(s) used AND whether they are one- or two-sided<br>*Only common tests should be described solely by name; describe more complex techniques in the Methods section.*
☐	☒	A description of all covariates tested
☐	☒	A description of any assumptions or corrections, such as tests of normality and adjustment for multiple comparisons
☐	☒	A full description of the statistical parameters including central tendency (e.g. means) or other basic estimates (e.g. regression coefficient) AND variation (e.g. standard deviation) or associated estimates of uncertainty (e.g. confidence intervals)
☒	☐	For null hypothesis testing, the test statistic (e.g. *F*, *t*, *r*) with confidence intervals, effect sizes, degrees of freedom and *P* value noted<br>*Give P values as exact values whenever suitable.*
☒	☐	For Bayesian analysis, information on the choice of priors and Markov chain Monte Carlo settings
☒	☐	For hierarchical and complex designs, identification of the appropriate level for tests and full reporting of outcomes
☒	☐	Estimates of effect sizes (e.g. Cohen's *d*, Pearson's *r*), indicating how they were calculated

Our web collection on statistics for biologists contains articles on many of the points above.

Software and code

Policy information about availability of computer code

Data collection	N/A
Data analysis	N/A

For manuscripts utilizing custom algorithms or software that are central to the research but not yet described in published literature, software must be made available to editors/reviewers. We strongly encourage code deposition in a community repository (e.g. GitHub). See the Nature Research guidelines for submitting code & software for further information.

Data

Policy information about availability of data

All manuscripts must include a data availability statement. This statement should provide the following information, where applicable:

- Accession codes, unique identifiers, or web links for publicly available datasets
- A list of figures that have associated raw data
- A description of any restrictions on data availability

All data generated or analyzed during this study are included in the published article (and its supplementary information files). All data are available from the authors upon reasonable request.

Field-specific reporting

Please select the one below that is the best fit for your research. If you are not sure, read the appropriate sections before making your selection.

☒ Life sciences ☐ Behavioural & social sciences ☐ Ecological, evolutionary & environmental sciences

Life sciences study design

All studies must disclose on these points even when the disclosure is negative.

Sample size	Sample sizes for mouse experiments were based on the experience of our group with these models (n=5 per group for the electrolytic model and n=10 for the stenosis model). We did not have preliminary data available for a power calculation.
Data exclusions	No data were excluded.
Replication	All experiments were performed at least three times, and all these data are presented in the manuscript; replicates were always performed on different days.
Randomization	Studies with patient neutrophils utilized consecutive patients recruited from the corresponding author's clinic. Mouse studies always utilized litter-mates for the experimental and control groups.
Blinding	Most data were agnostic in nature (i.e., plate-reader value). For mouse studies, thrombus size was determined by a surgeon who was blinded to the treatment conditions.

Reporting for specific materials, systems and methods

We require information from authors about some types of materials, experimental systems and methods used in many studies. Here, indicate whether each material, system or method listed is relevant to your study. If you are not sure if a list item applies to your research, read the appropriate section before selecting a response.

Materials & experimental systems

n/a	Involved in the study	
☐	☒	Antibodies
☒	☐	Eukaryotic cell lines
☒	☐	Palaeontology
☐	☒	Animals and other organisms
☐	☒	Human research participants
☒	☐	Clinical data

Methods

n/a	Involved in the study	
☒	☐	ChIP-seq
☒	☐	Flow cytometry
☒	☐	MRI-based neuroimaging

Antibodies

Antibodies used	Rabbit polyclonal antibody against neutrophil elastase (Abcam 21595)
Validation	The neutrophil-elastase antibody provided consistent "decoration" of NETs as in our extensive experience and publication with this antibody.

Animals and other organisms

Policy information about studies involving animals; ARRIVE guidelines recommended for reporting animal research

Laboratory animals	Mice were housed in a specific pathogen-free barrier facility, and fed standard chow. Male C57BL/6 mice were purchased from The Jackson Laboratory, and used at 8-10 weeks of age.
Wild animals	N/A
Field-collected samples	N/A
Ethics oversight	Experimental protocols were approved by the University of Michigan Institutional Animal Care and Use Committee.

Note that full information on the approval of the study protocol must also be provided in the manuscript.

Human research participants

Policy information about studies involving human research participants

Population characteristics	The patient population is described in detail (age, gender, disease duration, laboratory studies, clinical history, medications) in Supplementary Table 1.
Recruitment	Studies with patient neutrophils utilized consecutive patients recruited from the corresponding author's clinic.

Ethics oversight

This study was approved by the University of Michigan Institutional Review Board, and all participants provided informed consent for blood donation.

Note that full information on the approval of the study protocol must also be provided in the manuscript.
